# PMechDB: A Public Database of Elementary Polar Reaction Steps

**DOI:** 10.1021/acs.jcim.3c01810

**Published:** 2024-03-14

**Authors:** Mohammadamin Tavakoli, Ryan J. Miller, Mirana Claire Angel, Michael A. Pfeiffer, Eugene S. Gutman, Aaron D. Mood, David Van Vranken, Pierre Baldi

**Affiliations:** † Department of Computer Science, University of California, Irvine, Irvine, California 92697, United States; ‡ Department of Chemistry, University of California, Irvine, Irvine, California 92697, United States

## Abstract

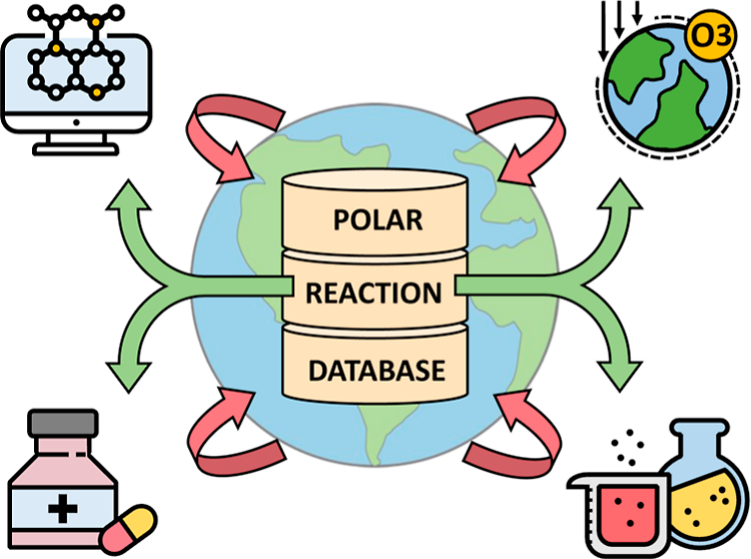

Most online chemical reaction databases are not publicly accessible or are fully downloadable. These databases tend to contain reactions in noncanonicalized formats and often lack comprehensive information regarding reaction pathways, intermediates, and byproducts. Within the few publicly available databases, reactions are typically stored in the form of unbalanced, overall transformations with minimal interpretability of the underlying chemistry. These limitations present significant obstacles to data-driven applications including the development of machine learning models. As an effort to overcome these challenges, we introduce PMechDB, a publicly accessible platform designed to curate, aggregate, and share polar chemical reaction data in the form of elementary reaction steps. Our initial version of PMechDB consists of over 100,000 such steps. In the PMechDB, all reactions are stored as canonicalized and balanced elementary steps, featuring accurate atom mapping and arrow-pushing mechanisms. As an online interactive database, PMechDB provides multiple interfaces that enable users to search, download, and upload chemical reactions. We anticipate that the public availability of PMechDB and its standardized data representation will prove beneficial for chemoinformatics research and education and the development of data-driven, interpretable models for predicting reactions and pathways. PMechDB platform is accessible online at https://deeprxn.ics.uci.edu/pmechdb.

## Introduction

The polar mechanism represents the most prevalent type of mechanism in organic and organometallic chemistry. These reactions are characterized by heterolytic bond cleavage and the formation of charged intermediates. Owing to the high reactivity of polar reactants, many such reactions can take place under standard and physiological conditions.^1−3^ Consequently, polar reactions are a subject of considerable interest and are widely employed in the fields of organic, inorganic, organometallic, biological, and environmental chemistry.^4,5^ As an illustration of their significance, the field of organocatalysis, acknowledged by the 2021 Nobel Prize in chemistry, is strongly dependent on polar mechanistic steps. Organocatalysis is used in medicinal chemistry^6^ and has been employed in the industrial production of pharmaceuticals.^7^

Machine learning methods, and particularly deep learning, are increasingly playing a central role in science and technology.^8^ In the domain of chemistry, considerable efforts have been made to develop computer models that can automate tasks such as drug discovery, organic synthesis, and molecular property predictions.^9−15^ Data-driven machine learning techniques necessitate vast chemical datasets for training, yet most of the currently available reaction databases are either not readily accessible,^16^ or contain noisy and incomplete data, as well as inaccurate or unlabeled information. These limitations make it challenging to train robust and accurate predictive data-driven models for chemoinformatics reaction problems. To partially address such limitations, we introduce PMechDB, a comprehensive and extensible database containing over 12,000 elementary polar reaction steps that have been manually curated. Additionally, the database contains more than 90,000 polar reactions generated through combinatorial methods using nucleophiles and electrophiles extracted from the Mayr-Ofial database. The reactions encompassed in PMechDB are manually curated and incorporate accurate reactive atom mappings, balanced reactants and products, intermediates, side products, and arrow-pushing mechanisms. This database is publicly available and is hosted on a web server through the DeepRXN Web site at https://deeprxn.ics.uci.edu/pmechdb. The Web site provides an interactive user interface that allows for searching specific reactions, filtering reactions based on their classification, displaying corresponding arrow-pushing mechanisms, downloading the current dataset, and uploading novel elementary reaction steps. The Web site allows users to draw individual elementary step mechanisms or provide csv files to upload many elementary step mechanisms concurrently. We emphasize the importance of dependable, accurately annotated, and readily expandable datasets for effectively training machine learning models in the field of chemistry. So, we invite users to contribute additional reactions to the PMechDB database, which will be curated, cleaned, and scrutinized by the PMechDB team of organic chemists. If deemed satisfactory, the reactions will be aggregated into the dataset, further enhancing its scope and reliability.

## Background

### Reaction Representation: Overall Transformation vs Elementary Steps

**Figure 1:**
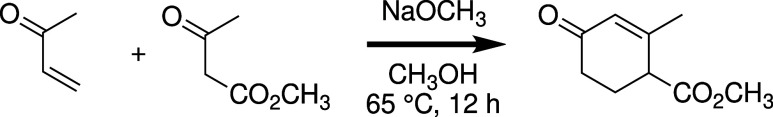
Robinson annulation is an important ring-forming reaction used by organic chemists. This is an example of a total transformation approach to representing the reaction. The final product is predicted from the reactants without any intermediate states or mechanistic steps.^17^

Overall transformations are popular and simple ways for organic chemists to represent a reaction. This representation consists of a set of reactant molecules that, when added together, form a set of product molecules, usually focusing on a single target, as seen in Figure 1. This representation contains no information about intermediate states or the stepwise mechanism of the reaction. Although this approach clearly describes the reactants and the products of a reaction, the limited amount of information makes it difficult for chemists to use these representations to reason about how the reaction proceeded or what the underlying driving forces were.

**Figure 2:**
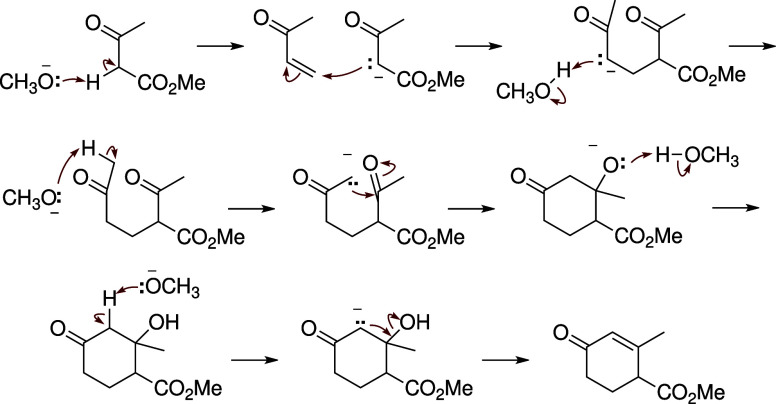
Example of an elementary step approach to represent the Robinson annulation. The final product is predicted from a series of elementary steps between intermediate molecules with arrow-pushing mechanisms.

The elementary step approach is a more complex representation that breaks the overall chemical transformations into a series of elementary steps, as seen in Figure 2. Each of these elementary mechanistic steps involves a single transition state. This series of elementary steps can then be concatenated together to represent the overall chemical transformation. By breaking reactions down into smaller mechanistic steps, organic chemists can gain insights into how these reactants will combine to form the resulting products and why they may combine in the way they do.

### Existing datasets of Elementary Reaction Steps

Existing databases, including Open Reaction Database (ORD), REAXYS, and SciFinder, have millions of reactions with information on reactants, products, conditions, and yields, but they only present reactions as overall transformations.^18^ Although AI models can learn from these data to predict chemical transformations and assist synthetic chemists, they do not offer much insights into the underlying mechanisms of chemical transformations. The PMechDB database, on the other hand, enables the training of models to predict arrow-pushing mechanisms involved in retrosynthetic planning, which offers a unique perspective on chemical reaction training data.

The most similar known database to PMechDB is the Radical Mechanistic Database.^19^ RMechDB is a publicly accessible database of 5500 elementary radical reaction steps which contains an interactive web interface for searching, downloading, and uploading the reactions. These elementary step radical reactions were manually curated by expert chemists from organic chemistry textbooks and atmospheric literature, including research articles and publications related to gas-phase radical processes. These elementary step reactions have been shown to enable the training and development of radical reaction predictors.^20^ Although this dataset is similar to PMechDB in structure, it is entirely composed of radical reaction steps, while PMechDB consists of polar reaction steps. Polar reactions are used everywhere in organic chemistry, and understanding these mechanisms is fundamental to developing ML models that are useful to synthetic chemists. Additionally, PMechDB contains significantly more reactions than RMechDB (∼100,000 compared to ∼5500) and includes updates to the interactive web interface.

To our knowledge, the only other database of reaction mechanisms is supported by reaction mechanism generator (RMG).^21^ RMG is a quantitative approach to predict mechanistic pathways by (a) evaluating the rate constants of the possible competing elementary steps of the reaction; (b) determining the rate ratio of the steps by plugging in concentrations of the reactants into the rate law of each elementary step.

For thermochemical calculations, the RMG mainly uses Benson group increment theory (BGIT).^21^ However, since the BGIT fails to properly describe ring strains and noncovalent interactions,^22−25^ for cyclic species, RMG carries out geometry optimization with molecular mechanics and subsequent single-point calculation with advanced quantum mechanical methods to derive the necessary thermodynamic parameters. To approximate kinetic parameters, RMG generates reaction pathways using a predetermined and extensible set of reaction families and assumes that reactions between similar reacting sites in a family will have similar rates.^21^ The original RMG database focuses on constructing mechanisms for mainly radical reactions with species involving carbon, hydrogen, oxygen, sulfur, and nitrogen.^21^

Our work aims to create a database that better represents typical polar reaction mechanisms found in organic chemistry research. Rather than focusing on a limited group of reactions with quantitatively estimated rate constants, our database represents feasible and manually verified polar reactions found in organic chemistry textbooks and the organic chemistry literature. Our hope is that by training on a dataset of elementary steps found in literature, AI models can be trained to predict arrow-pushing mechanisms for reactions that are more relevant to retrosynthetic planning.

## PMechDB: Underlying Dataset

### Manually Curated Dataset

**Figure 3:**
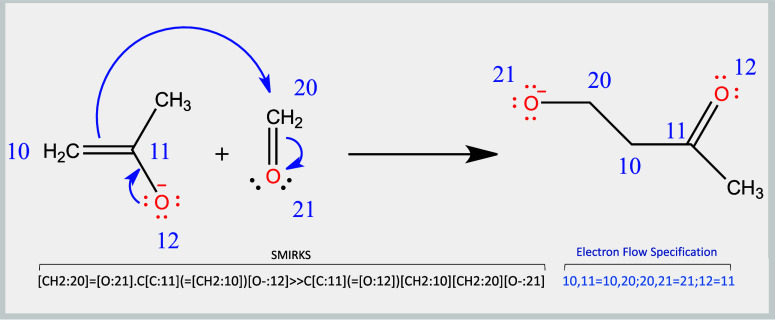
PMechDB format for depicting elementary steps using SMIRKS strings with an electron flow specification.

The primary dataset comprises 12,799 SMIRKS, each accompanied by an electron flow specification conveyed by curved arrows (Figure 3). Each SMIRK represents a plausible elementary reaction step that corresponds to a single transition state that can be portrayed through Lewis structures and curved arrows. It should be noted, however, that applying curved arrows to nontraditional bonds, such as hydrogen bonds, dative bonds, or molecular interaction representations depicted through dotted/dashed lines, can lead to incorrect formal atomic charges. To avoid such inaccuracies, and maintain consistency with the arrow-pushing convention, the dataset employs widely accepted, one-step curved arrow representations of some processes that are known to involve noncovalent intermediates such as example proton transfers^26^ and *S*_*N*_2 displacements.^27,28^ About 4% of the entries are resonance interconversions, but it is worth noting that these resonance steps do not possess a transition state. To the best of our knowledge, curved arrow mechanistic steps are not used to reveal temperature effects, concentration effects, implicit solvents, or roles of spectator species. The use of Lewis structures and arrow-pushing is considered to be a valuable compromise between generality and trainability versus precise chemical accuracy. Before adding any of these mechanisms to the PMechDB dataset, our organic chemistry team manually looked through each mechanism and deemed these steps as both plausible and elementary.

The dataset originated with 2989 polar reaction steps derived using Reaction Explorer, a rule-based system,^29^ and it was later expanded to 5551 polar reaction steps to train reaction predictor.^30^ To add these additional polar reactions, the reaction explorer was expanded to include more atom types such as sulfur, phosphorus, and magnesium, and reactions were manually curated from graduate-level textbooks to improve the coverage of the dataset and introduce novel reaction types.^31^ Subsequently, the existing dataset underwent a curation process to eliminate redundant entries and steps deemed impractical under standard laboratory conditions (e.g., <150 °C). Eventually, the dataset was expanded to over 12,000 entries through a series of iterative training and testing cycles of reaction predictors. Additional elementary reaction steps were sourced from undergraduate- and graduate-level organic chemistry course material, research presentations, and primary research literature. Any gaps in training were identified based on the presence of implausible steps in the top-ranked predictions.

A major challenge in developing the PMechDB was determining the plausibility of single-step mechanisms. In many cases, spectroscopic techniques such as mass spectrometry, NMR, and IR can be employed to describe published products. As a result, organic transformations in databases such as REAXYS, SciFinder, and ORD can be readily confirmed. However, validating mechanistic steps that involve a single transition state is a more challenging. Typically, electronic structure computations or time-consuming experimental techniques, including chemical kinetics, isotopic labeling, and crossover experiments, are necessary for experimental verification of a mechanistic step. It is often stated that mechanisms can be refuted but never proven. To assist scientists in constructing mechanistic pathways and predicting the outcomes of organic reactions, we aimed to develop a dataset containing plausible fundamental reaction steps from an organic chemist’s perspective.

In PMechDB, the plausibility of a mechanistic step is evaluated subjectively by our organic chemistry team based on its likelihood of occurrence. In instances where multiple pathways have been proposed in the literature, and there is discordance between them, it is recommended to incorporate steps from all potential pathways into the dataset. This approach ensures that any suggested pathway utilizing the data reflects the uncertainty present in the literature.

**Figure 4:**
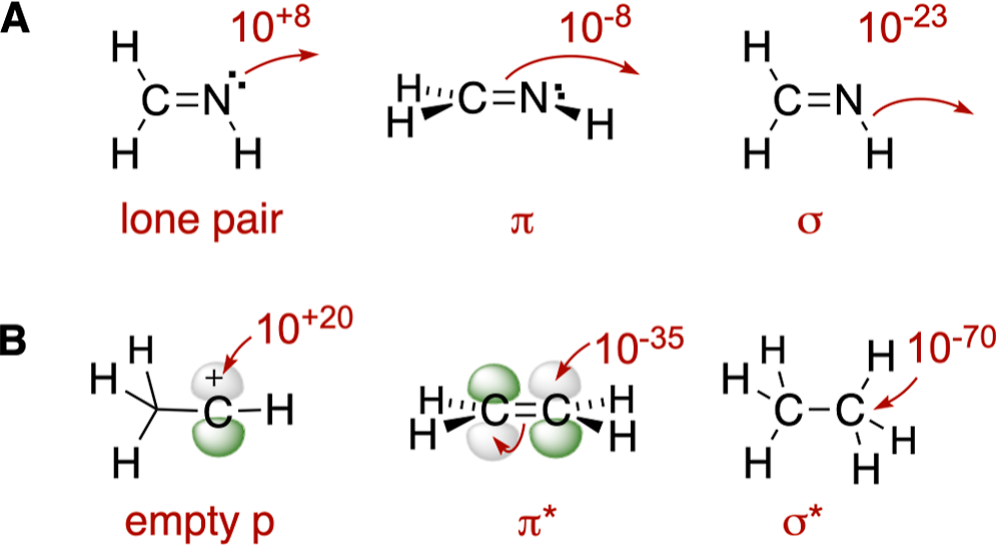
(A) Three classes of filled frontier orbitals and (B) three classes of empty frontier orbitals. Numbers correspond to methyl ion affinities.

To further organize the data, each mechanism was classified by an orbital interaction pair. Based on a system with three types of filled orbitals interacting with three types of unfilled orbitals, there are nine (3 × 3) categories of elementary arrow-pushing steps as seen in Figure 4.

**Figure 5:**
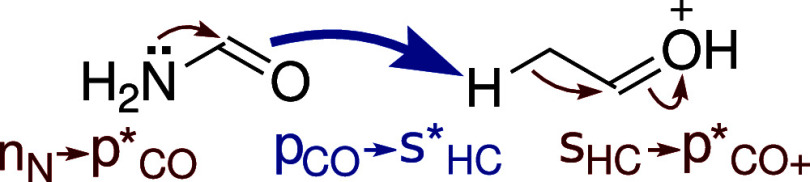
Chains of canonical orbital interactions.

The complexity of molecular species in the core dataset of PMechDB ranges from simple species like those in RMG to structurally complex molecules found in ORD, REAXYS, and SciFinder. It is not uncommon for a curved arrow depiction of an elementary reaction step to involve chains of frontier orbital interactions. For example, the enolization depicted in Figure 5 represents three sequential types of orbital interactions: (1) donation of *n*_N_ into π_CO_*, (2) donation of π_CO_ into σ_HC_*, and (3) donation of σ_HC_ into π_CO^+^_^*^. Orbital interactions (1) and (3) represent intramolecular arrows, while orbital interaction (2) represents an intermolecular arrow. To classify each reaction, we use the intermolecular orbital interaction; therefore, the example above would be classified according to the blue arrow as a pi_sigma* reaction.

**Figure 6:**
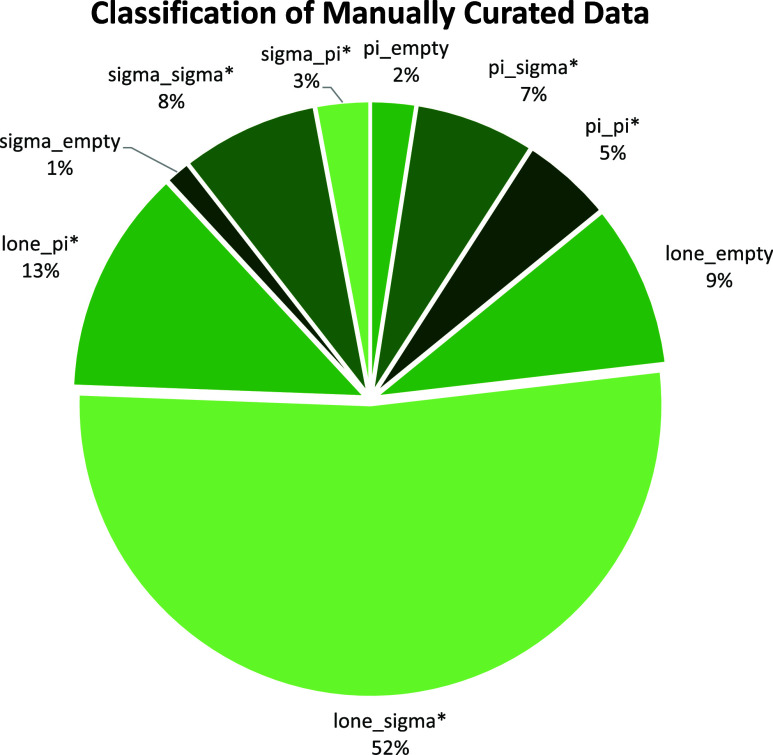
Classification of manually curated reactions using 9-class orbital interaction pairs.

A more detailed explanation of the nine categories of orbital interactions can be viewed in the Supporting Information. The distribution of reactions across the nine categories of orbital interaction, as classified by manual curation, is presented in Figure 6. There are far more entries in the category of lone pair adding to σ*, reflecting the importance of proton transfer steps in stepwise polar reaction mechanisms.

### Combinatorial Elementary Steps Based on the Mayr Nucleophiles and Electrophiles

To supplement the core set of over 12,000 highly diverse polar steps, polar reaction steps were assembled from combinatorial pairs of electrophiles and nucleophiles from the data in the Mayr-Ofial database of nucleophilicity (*N*, *s*_N_) and electrophilicity parameters (*E*).^32^ For nucleophiles with multiple entries in different solvents, only a single entry was chosen, but parameters determined in protic solvents were excluded. Hindered electrophiles and nucleophiles with steric dependences were excluded. Some hard anionic nucleophiles such as acetate, benzoate, *p*-nitrobenzoate, 3,5-dinitrobenzoate, DMSO (O attack), and methyl carbonate were excluded. 853 (out of 1254) Mayr nucleophiles and 313 (out of 352) Mayr electrophiles were selected leading to 257,712 nucleophile–electrophile combinations. For five-star reference electrophiles and nucleophiles, the diffusion limit, *s*_N_(*N* + *E*) provides a good approximation of log  *k*_20°C_ but the equation is less accurate for other entries.^33,34^ In total, 96,558 nucleophile–electrophile combinations with *s*_N_(*N* + *E*) ≥ 3 were translated into mapped SMIRKS with electron flow specification. One thousand of the least reactive combinations were manually checked to confirm plausibility. *S*_*N*_2 reactions are of seminal importance in organic chemistry, but iconic sp^3^ electrophiles, such as alkyl halides, were notably absent from the Mayr database.

**Figure 7:**
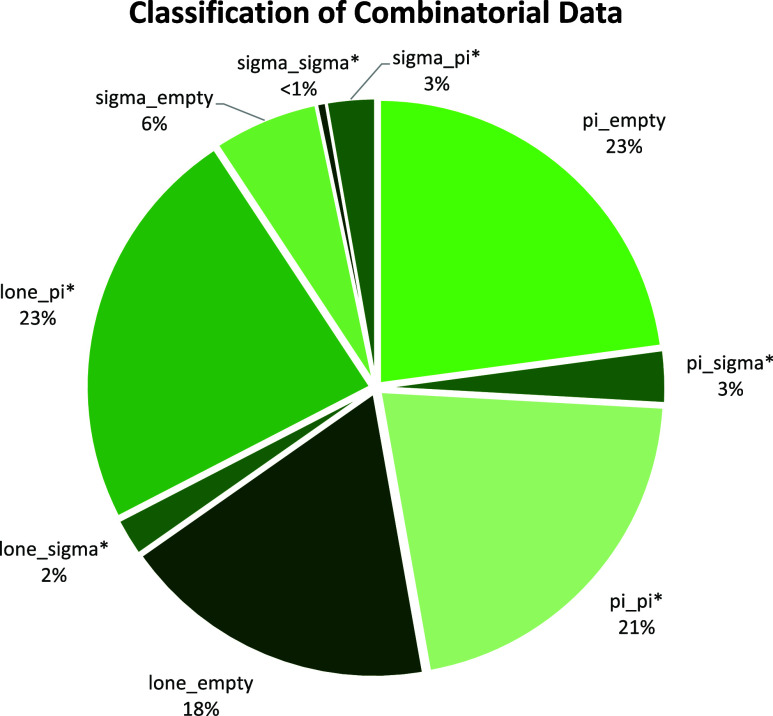
Classification of combinatorial reactions using 9-class orbital pair interactions.

Categorizing the combinatorial data into the nine categories of orbital interaction, we have the following distribution of reactions as seen in Figure 7.

### Train and Test Splits

**Figure 8:**
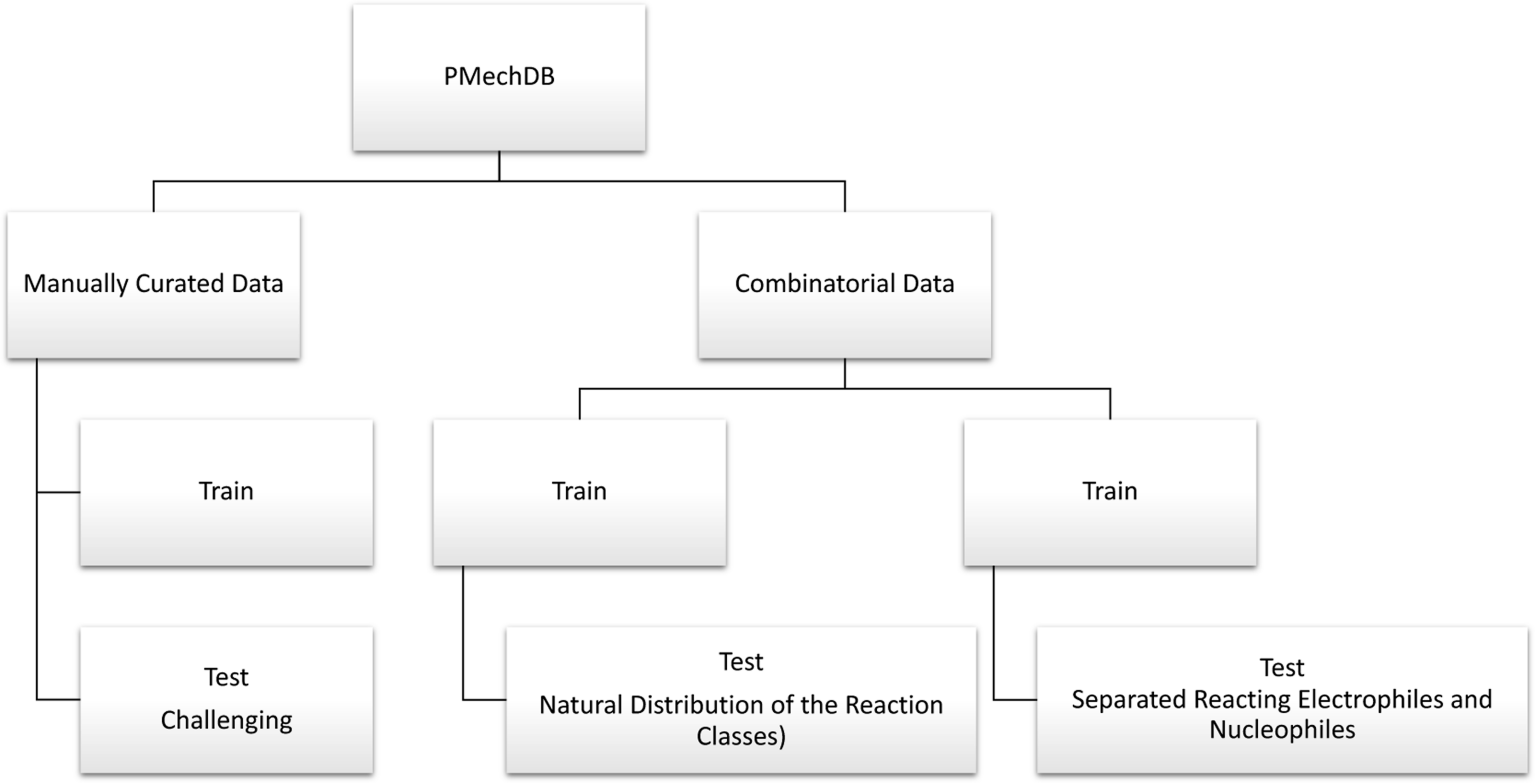
Train and test splits are provided by PMechDB.

PMechDB is primarily designed to serve as a reliable source of training and evaluation data for the development of machine learning models. Standard splits of training and evaluation data are provided to facilitate the development and comparison of these models. We offer standard data splits for both manually curated and combinatorial mechanistic reactions. For manually curated mechanistic reactions, we have compiled a set of 300 challenging reactions meticulously selected by expert chemists. These reactions are intended to assess the generalization capabilities of machine learning models for large and complex reaction systems. We refer to this test dataset as the challenging test split. For the combinatorial dataset, we offer two standard train and test splits. First, a 90/10 split with a training set containing 86,303 reactions and a test set containing 9585 reactions. The test reactions are sampled in such a way that they contain the same proportion of the nine reaction classes as the training set (Figure 7). This train and test split enables the evaluation of the performance of predictive models across each of the nine polar reaction classes. However, due to the nature of the combinatorial data, many of the reacting electrophiles and I have nucleophiles obtained from the Mayr reactivity table that may appear in both the train and the test sets. Therefore, a second split is constructed by initially partitioning the reacting electrophiles and nucleophiles into train and test sets and then performing the combinatorial generation. This train and test split is designed to minimize the overlap of reacting functional groups between the train and test data. Thus, this split enables the evaluation of the generalization capability of predictive models for unseen reacting groups. As the electrophiles and nucleophiles are split, the total number of combinatorial reactions in the second split is smaller, resulting in a total of 54,048 train reactions with 6093 test reactions. Our preliminary experiments indicate that achieving high test performance on the second split is more challenging, yet models trained on this split demonstrate greater generalization capability and less overfitting. The structure of PMechDB, along with the train and test data splits, is illustrated in Figure 8. In the Supporting Information, additional details are presented concerning the training and testing data splits for both manually curated and combinatorial datasets. This information encompasses distributions of molecular weights, atom types, and numbers of atoms in elementary step reactions.

### Database Schema

The database is implemented using the PostgreSQL^35^ database management system,^36^ to store, query, and retrieve reaction instances both efficiently and safely. We use OpenEye Scientific Software^37^ toolkits OEChem,^38^ and OEDepict^39^ for chemoinformatics processing and depiction. In addition, we use Chemaxon Marvin^40^ for displaying and characterizing chemical structures, substructures, and steps with their corresponding arrow-pushing mechanisms.

The PMechDB database schema comprises three fundamental models: (1) Reaction, (2) Molecule, and (3) Atom. The inter- and intraintegration of these three models allow for fast and efficient reaction search and retrieval. As the naming suggests, each elementary step is stored as an instance of the reaction model which comes with several descriptive fields. These fields are designed to uniquely represent an elementary step reaction and all of the available metadata associated with it. Here, we list the main fields of the reaction model.

1. **Reaction ID:** Each reaction is associated with a unique ID number.
2. **Canonicalized atom mapped SMILES of the reactants:** The reactant molecules are represented by SMILES strings, with the inclusion of integer labels denoting the atoms involved in the reaction. A standardized labeling convention is employed, whereby the participating atoms located in the nucleophile component are assigned labels commencing at 10 and sequentially increasing by one for each subsequent atom, while the participating atoms located in the electrophile component are assigned labels commencing at 20 and also sequentially increasing by one for each subsequent atom.
3. **Canonicalized atom mapped SMILES of the products:** The unique SMILES representation of the product molecules generated from the reactive reactants with atom mappings.
4. **Canonicalized arrow codes:** The standard codes for arrow pushing mechanisms contain the integer labels of the participating atoms on the reactants side. The standard arrow codes begin from the integer label (starting at 10) on the nucleophilic group.
5. **Spectator molecules:** The unique SMILES representation of the molecules that are present in the reaction but not participating in the electron transfer.
6. **Source:** The SMILES string of the reactant molecule with the source (nucleophilic) reactive atom marked.
7. **Sink:** The SMILES string of the reactant molecule with the sink (electrophilic) reactive atom marked.
8. **Dataset:** The dataset where the polar reaction belongs to, either “manually curated” or “combinatorial”.
9. **Orbital Pair Classification:** The orbital pair class belonging to the nine categories of orbital interaction outlined in the Supporting Information.
10. **Date of Insertion:** The date and time when the reaction was inserted into the database.

Given the fields associated with the Reaction model, an instance of this model in PMechDB can be uniquely retrieved from the database using either the **Reaction ID** or the combined properties 2–5 as the key.

The Molecule model has three fields corresponding to the unique molecule ID, canonicalized SMILES string of the molecule, and the OEChem MolBase object.^38^ An instance of the Molecule model has a many-to-many relation with the reactant molecules, product molecules, and spectator molecules fields of the reaction model.

The Atom model has five fields corresponding to the unique ID, canonicalized atom mapped SMILES string of the parent molecule, a boolean for if the atom can act as a source, a boolean for if the atom can act as a sink, and the OEChem AtomBase object.^38^ An instance of the Atom model has a many-to-many relation with the source orbital and sink orbital fields of the reaction model.

**Figure 9:**
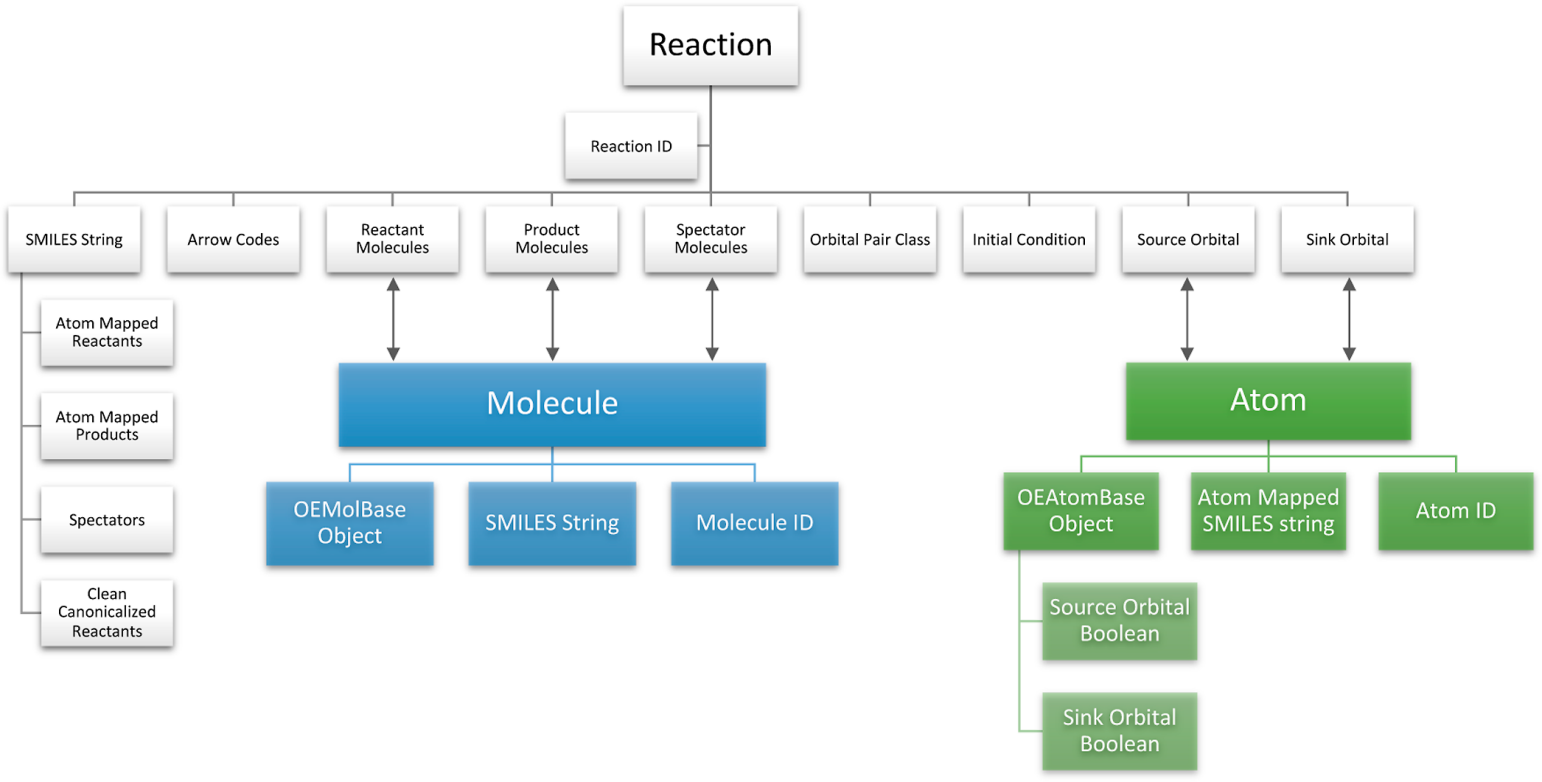
Relations of the three fundamental models of the PMechDB database. The arrows represent many-to-many relations.

The schema with the fields described in Figure 9 is designed not only to provide efficient storage and retrieval but also to enable the automated population of the fields for new steps that are contributed to PMechDB by the community, as described in the section on Uploading New Data.

## PMechDB: Web Server

The web server of the PMechDB offers three interfaces for (1) searching the data; (2) downloading the data; and (3) uploading new data.

### Searching the Data

The PMechDB search interface is accessible at https://deeprxn.ics.uci.edu/pmechdb/rsearch. This interface offers a user-friendly means of searching through the expansive dataset via two search methods: (1) reaction search, where the search entity is a chemical reaction, and (2) compound search, where the search entity can be a molecule, a substructure, or an atom. The capabilities of the search interface allow for tailored filtration of the database based on a variety of reaction attributes such as the reaction category (e.g., manually curated vs combinatorial) or the 9-class classification of polar reactions (e.g., lone-empty).

### Reaction Search

1. Exact search: Using this method, the user can search through the database for specific chemical reactions with known reactants and products. The user is required to input the query in the form of the SMIRKS of an elementary step, specifying the reactants and products, but not the arrow code. The system then searches and displays all elementary steps with the same reactants and products as the query but with additional molecules involved as reagents or spectators.
2.Similarity search: Using this method, the user can find the reactions in PMechDB that are most similar to an input reaction. The user is required to input the query as the SMIRKS of an elementary step, specifying the reactants and products, and the desired number of similar reactions (N) to be retrieved. Upon searching, the system displays N elementary steps sorted from the most similar to the least similar to the input query. The current version of RMechDB is equipped with the following similarity metrics computed on various representations of the elementary steps:
  (a) The Tanimoto, dice, and cosine distance between the binary Extended Connectivity Fingerprints (ECFP)^41^ of the elementary steps.
  (b) The Euclidean distance between the embedding of the elementary steps derived using a pretrained transformer architecture, trained on the SMIRKS of the USPTO dataset.^42,43^

### Compound Search

1. Molecule search: Using this method, the user can search through the PMechDB database for reactions containing specific molecules. The user is required to input a SMILES string containing the desired reactant and product molecules. Each molecule is separated by the “.” character, and reactants are separated from products by the “≫” character. If users want to search for reactants only, they may specify “≫” after the list of molecules. If users want to search for products only, they may specify “≫” before the list of molecules. If “≫” is omitted, the search will look for reactions where the molecule is contained on both sides of the reaction. After validating the SMILES input, the platform displays all elementary steps in its database that contain the specified molecules.
2. Reactive atom (molecular orbital) search: Using this method, the user can search the PMechDB database for reactions with specific atoms acting as the electron donor or electron acceptor. The user is required to input the atom-mapped SMILES string of a molecule and label the reactive atom using an integer between 1 and 11, while the other atoms are not labeled. The platform displays all elementary steps in its database where the labeled atom acts as one of the two main reactive atoms.
3. Substructure search: Using this method, the user can search the PMechDB database for reactions containing specific substructures. The user is required to input a SMARTS string containing the desired reactant and product substructures. Each substructure is separated by the “.” character, and reactants and products are separated by the “≫” character. The same search rules apply as mentioned in the molecule search section for searching for reactants/products only. Each substructure provided must be chemically valid. PMechDB displays all elementary steps in its database containing molecule(s) with the input substructures.

### Downloading the Data

**Figure 10:**
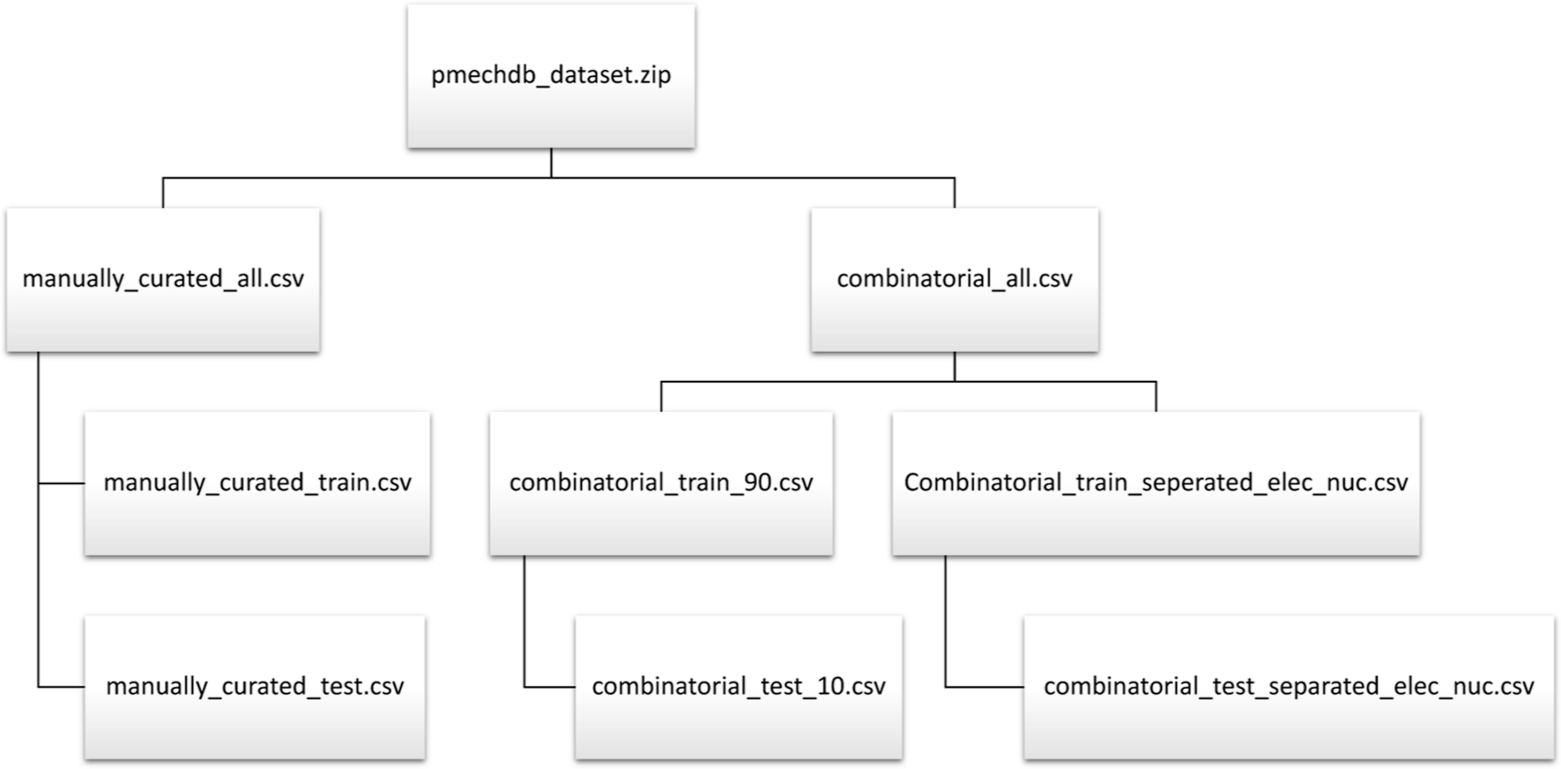
Name of the files within the downloaded PMechDB dataset.

The PMechDB chemical reaction dataset can be downloaded at the web address https://deeprxn.ics.uci.edu/pmechdb/download. The database is governed by the Creative Commons Attribution-NonCommercial-NoDerivs (CC-BY-NC-ND) license, which restricts its free public utilization solely to noncommercial purposes. This license prohibits alteration or redistribution of the dataset without proper citation of the original source. Upon agreement to the license terms and submission of personal information such as name, email, and institutional affiliation, users will receive an email containing several comma-separated values (CSV) files that encompass the entirety of the database, including metadata for both the manually curated and combinatorial reaction data. The structure of the downloaded dataset and the name of each file are shown in Figure 10.

### Uploading New Data

As we continue to expand the database of PMechDB, we extend an invitation to the scientific community to contribute novel polar elementary steps. Uploading new data in the form of polar mechanistic reactions can be done at: https://deeprxn.ics.uci.edu/pmechdb/upload. Contributing users are required to complete a submission form consisting of three fields: (1) the SMIRKS notation of the elementary step, (2) the corresponding electron flow specification, and (3) the source of the elementary step. Additionally, there exists an optional field where the user can provide Supporting Information about the reaction. Following submission, the proposed elementary step will undergo automated checks for validity and duplication followed by a comprehensive evaluation by our team of proficient organic chemists to ensure plausibility before its assimilation into the database. We follow the same verification process as it was introduced in.^19^ Finally, users can choose to upload reactions individually or as a large group formatted as a comma-separated-value file. More instructions can be found at https://deeprxn.ics.uci.edu/pmechdb/howtouse.

## Conclusions

PMechDB is a new platform for curating and sharing polar chemical reaction data. This platform addresses the limitations of existing databases by storing reactions in the form of canonicalized and balanced elementary steps with accurate atom mapping and arrow-pushing mechanisms. PMechDB contains over 100,000 elementary step reactions and is publicly accessible through its web interface. We postulate that this standardized representation and support for the public availability of reliable data will benefit research in chemoinformatics and the development of data-driven and predictive models. A database of elementary reaction steps can also be used in chemical education in different ways, for instance in combination with interactive tools for learning chemical reactions.^44,45^ PMechDB is a significant step toward improving the accessibility and usability of chemical reaction data, and we hope that it will inspire further developments in this field.

## Supporting Information Available

Additional statistics about the atom types, number of atoms in the reactions of each dataset, and detailed description of the classification scheme for polar elementary steps into nine classes (PDF)
