# Supplementary material for: PMechDB: A Public Database of Elementary Polar Reaction Steps
Source: J Chem Inf Model. 2024 Mar 14;64(6):1975–83. doi: 10.1021/acs.jcim.3c01810 (PMC10966657; doi:10.1021/acs.jcim.3c01810)
Supplement: Supplementary file 1 — ci3c01810_si_001.pdf [file ci3c01810_si_001.pdf]

Supporting Information

PMechDB: A Public Database of Elementary Polar Reaction Steps

Mohammadamin Tavakoli,[†] Ryan J. Miller,[†] Mirana Claire Angel,[†] Michael A. Pfeiffer,[‡] Eugene S. Gutman,[‡] Aaron D. Mood,[‡] David Van Vranken,[*,‡] and Pierre Baldi[*,†]

†*Department of Computer Science, University of California, Irvine, Irvine, California 92697, United States*

‡*Department of Chemistry, University of California, Irvine, Irvine, California 92697, United States*

E-mail: david.vv@uci.edu; pfbaldi@uci.edu

Additional Statistics on the Train and Test Splits

Molecular Weights

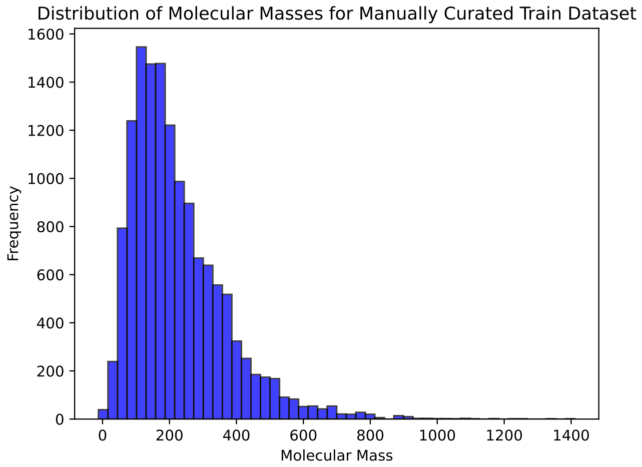

Figure S1: The distribution of molecular weights for the molecules in the manually curated training dataset.

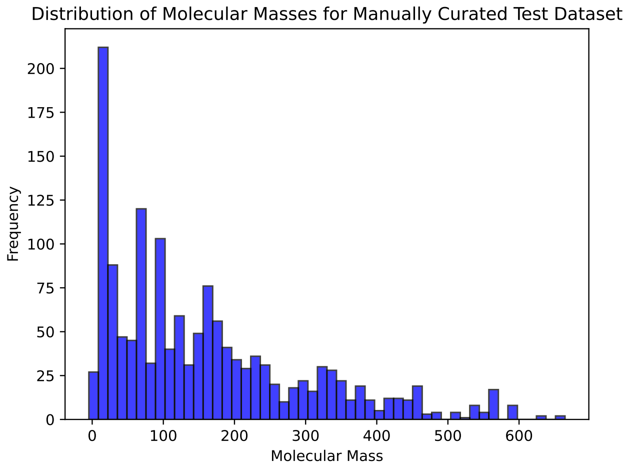

Figure S2: The distribution of molecular weights for the molecules in the manually curated testing dataset.

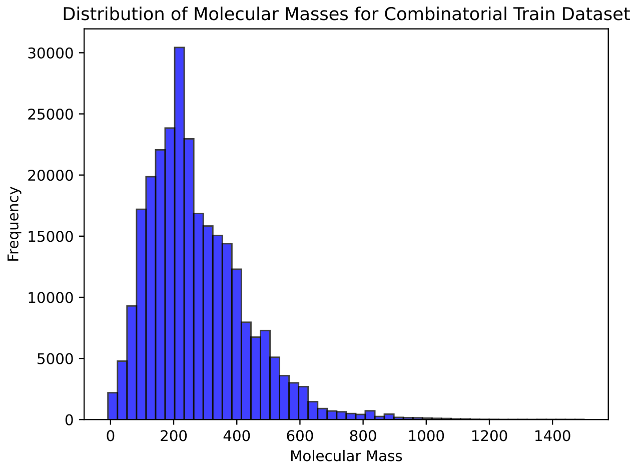

Figure S3: The distribution of molecular weights for the molecules in the non-separated combinatorial training dataset.

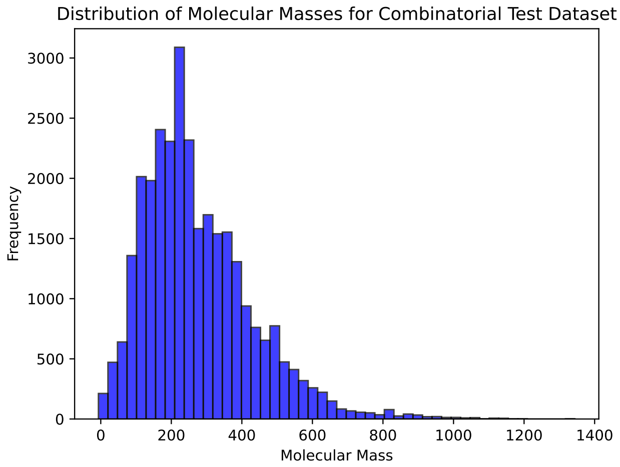

Figure S4: The distribution of molecular weights for the molecules in the non-separated combinatorial testing dataset.

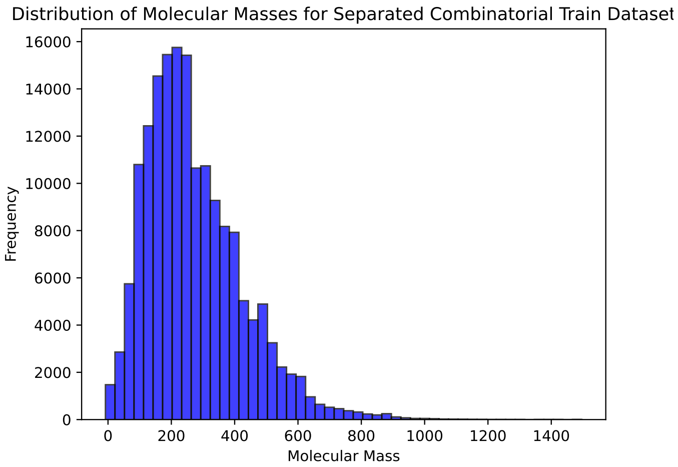

Figure S5: The distribution of molecular weights for the molecules in the separated combinatorial training dataset.

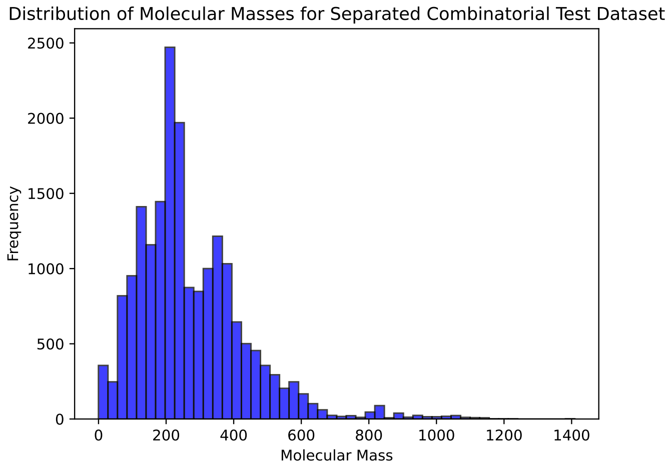

Figure S6: The distribution of molecular weights for the molecules in the separated combinatorial testing dataset.

Number of Atoms

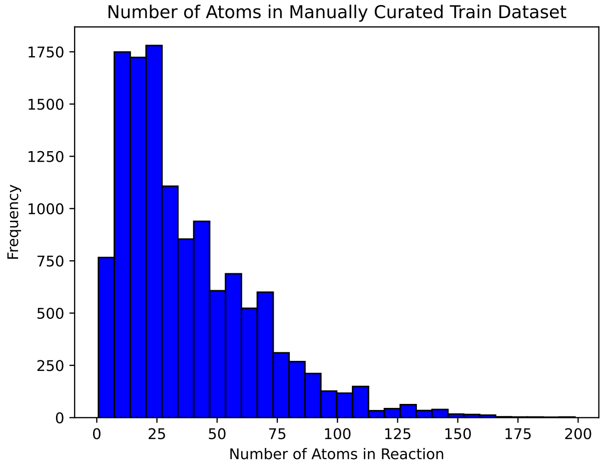

Figure S7: The distribution of the total number of atoms contained in each reaction for the manually curated training dataset.

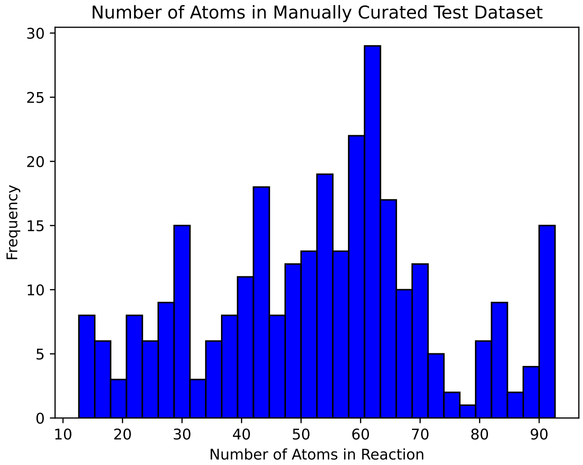

Figure S8: The distribution of the total number of atoms contained in each reaction for the manually curated testing dataset.

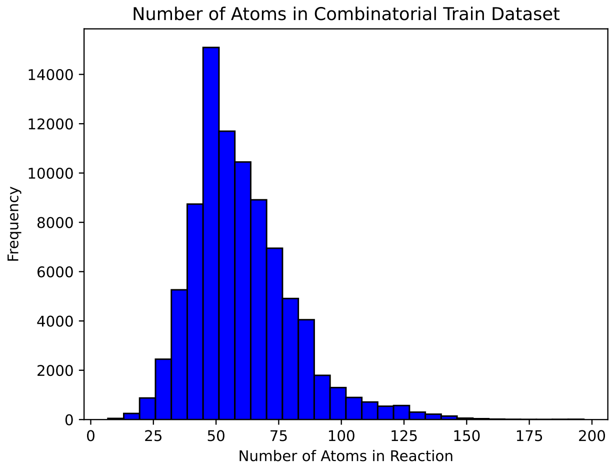

Figure S9: The distribution of the total number of atoms contained in each reaction for the non-separated combinatorial training dataset.

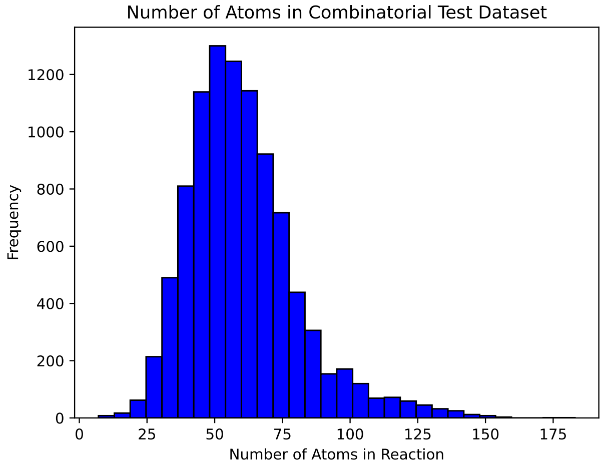

Figure S10: The distribution of the total number of atoms contained in each reaction for the non-separated combinatorial testing dataset.

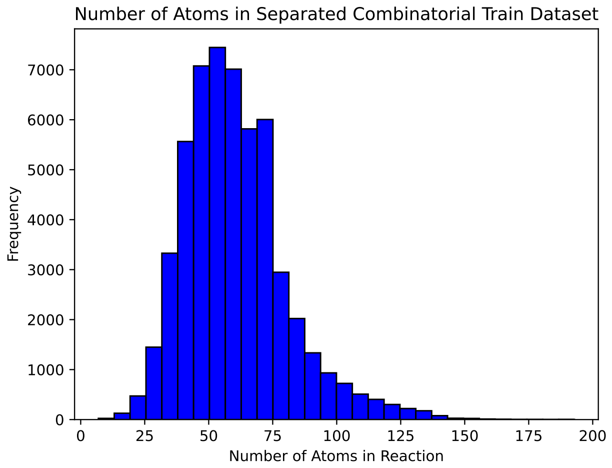

Figure S11: The distribution of the total number of atoms contained in each reaction for the separated combinatorial training dataset.

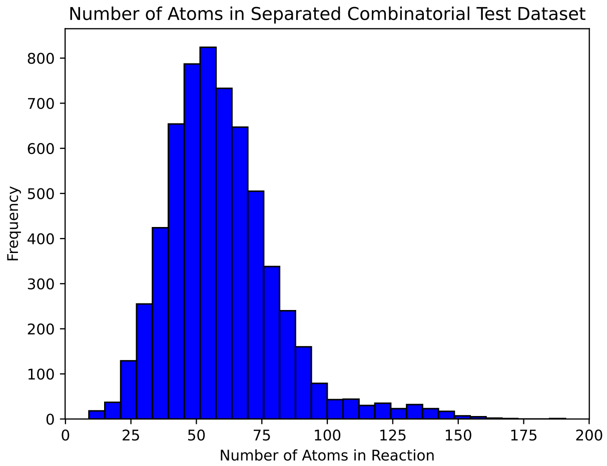

Figure S12: The distribution of the total number of atoms contained in each reaction for the separated combinatorial testing dataset.

Atom Type Frequency

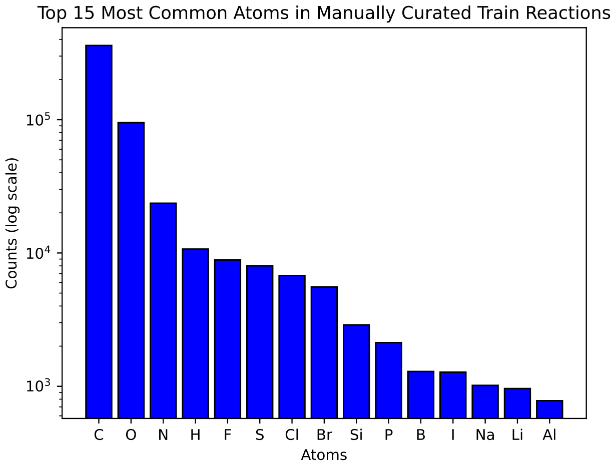

Figure S13: The distribution of atoms for the reactions in the manually curated training dataset.

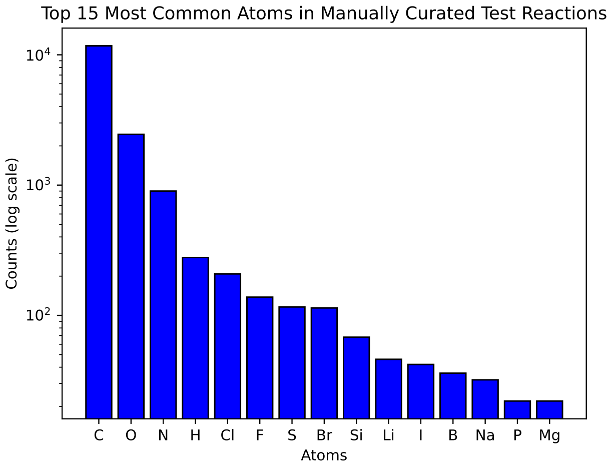

Figure S14: The distribution of atoms for the reactions in the manually curated testing dataset.

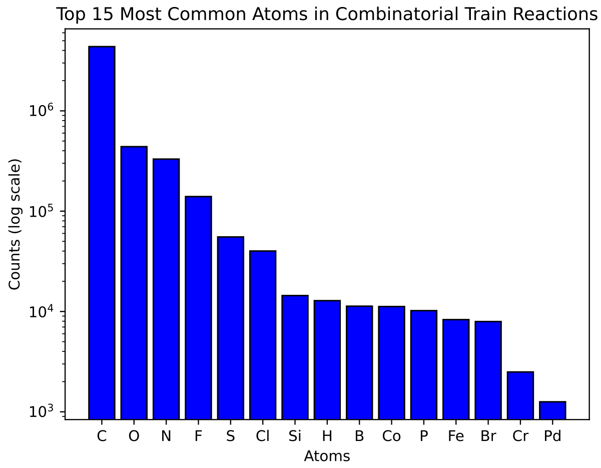

Figure S15: The distribution of atoms for the reactions in the non-separated combinatorial training dataset.

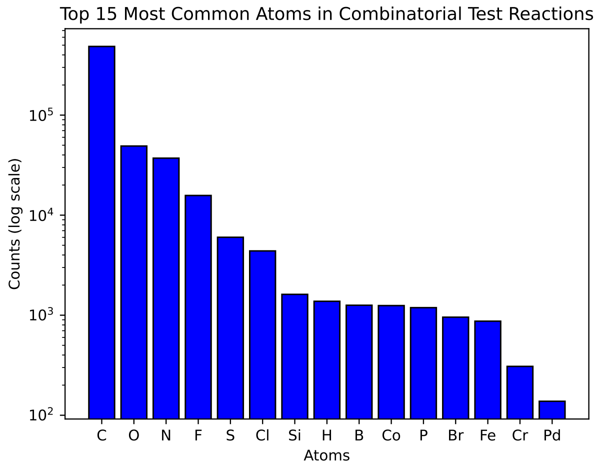

Figure S16: The distribution of atoms for the reactions in the non-separated combinatorial testing dataset.

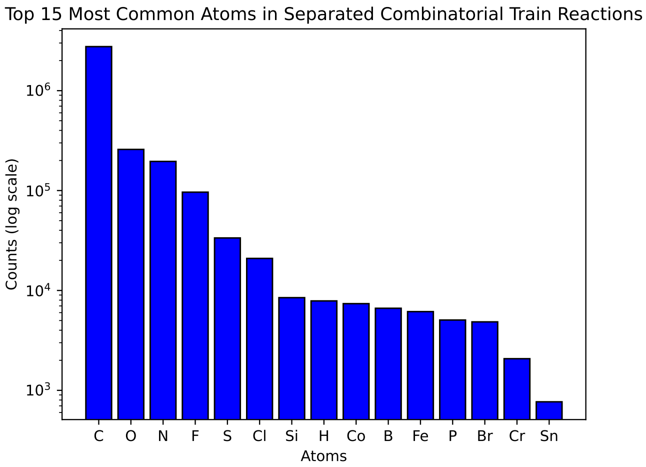

Figure S17: The distribution of atoms for the reactions in the separated combinatorial training dataset.

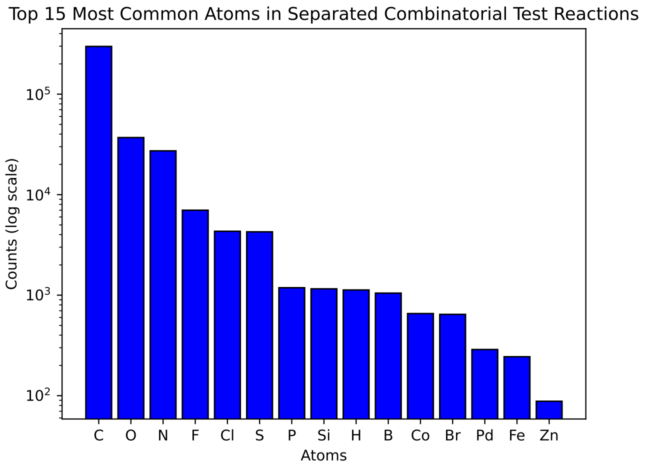

Figure S18: The distribution of atoms for the reactions in the separated combinatorial testing dataset.

Classification of Polar Elementary Step Reactions

We present comprehensive details on the classification of polar elementary steps into nine classes. These classes are defined by the interaction between three distinct source orbitals and three different sink orbitals. In each section, we begin by identifying the source orbital and subsequently describe the three potential interacting sink orbitals.

I. Source: Non-bonding Lone Pairs of Electrons

Non-bonding lone pairs on heteroatoms are among the most common types of frontier orbitals in curved arrow mechanisms. More than half of the 12,684 manually curated training steps involve non-bonding lone pairs adding to sink atoms. For arrow-pushing mechanisms, it is common to misrepresent alkali species such as HO–Na and H3C–Li as charge-separated ion pairs (Fig. S19A) even though a more realistic representation would involve covalent bonds between oxygen and metal (Fig. S19B).[1] About two-thirds of the Na+ -OR nucleophiles were depicted as charge-separated ionic species and the remaining third were depicted with a Na–OR bond. Any monomeric representation is an over-simplification of the complex oligomerization state (Fig. S19C) of alkali metal species.[2] This initial dataset contains strictly monomeric representations (ionic or covalent) of alkali metal species but could be readily expanded to include complex oligomeric representations of alkali metal aggregates as long as the structures have correct formal charges obeying the Lewis formalism. Neither dative bonds nor dashed hydrogen bonds obey the Lewis formalism and are incompatible with curved arrow mechanisms.

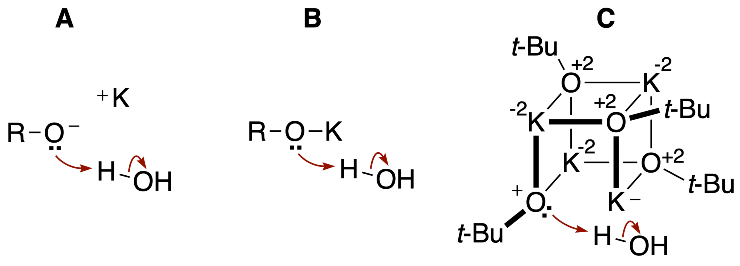

Figure S19: Increasingly accurate Lewis representation of alkali metal alkoxides: A. Monomeric, ionic, B. Monomeric, covalently bonded, C. Oligomeric, covalently bonded.

A. Lone Pair Adding to Empty Orbital

About one-tenth of the initial dataset contains steps involving the addition of non-bonding lone pairs to empty orbitals without displacement of a leaving group (Fig. S20). About half of these steps involve alcohol oxygens as source atoms and a fourth involves anionic alkoxide oxygens, with smaller numbers of heteroatom and halide anion nucleophiles. The empty orbitals are more diverse. The largest category of empty orbital acceptors for lone pairs are carbenium cations, corresponding to the first step in an S_N1 substitution mechanism. About a fourth of the steps in this category involve the addition of hard nucleophiles to silicon to form pentavalent siliconate intermediates, which is the first mechanistic step in silyl transfer processes. The third most common type in this category involves an addition to trivalent boron and aluminum, e.g., coordination of Lewis acids like BF_3 and $AlCl_3$, organoboron chemistry, hydridoaluminate reductions, and boron ester exchange. Associative processes with metals (e.g., RLi, Li^+, CuX_2, MgX_2, ZnX_2, TiX_4) were also present in this category.

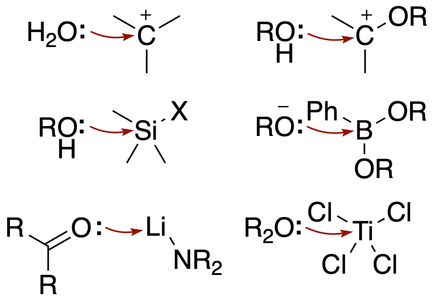

Figure S20: Examples of non-bonding lone pairs adding to empty orbitals.

There are many cases in which an electrophile with an empty p orbital can be alternately represented as an electrophile with a π^* orbital. For example, an oxocarbenium ion can also be represented as an oxonium C=O* π^* acceptor (Fig. S21). In most cases only one of the two types of representations are present in the dataset, without consistency, mirroring the lack of a convention in the field of organic chemistry.

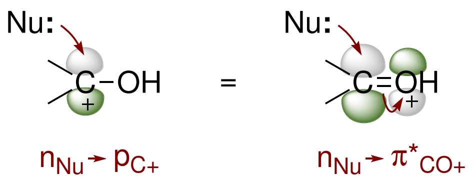

Figure S21: Two equivalent arrow-pushing depictions for addition to an oxocarbenium ion.

B. Lone Pair Adding to π^*

About a tenth of the initial dataset involves the addition of lone pairs to π^* orbitals (Fig. S22). About two-thirds of the lone pairs adding to double and triple bonds double and triple bonds were on neutral oxygen, neutral nitrogen, and anionic oxygen atoms. About a tenth of the training steps involved the addition of carbanion nucleophiles rendered with nucleophilic lone pairs. The most common π^* acceptor for lone pairs was a neutral carbonyl C=O followed by oxonium C=O$^+$ and iminium C=N$^+$. Over 10% of the steps in this category involved addition to conjugated pi acceptors. Only 3% of the mechanistic steps in this category involved the addition of lone pairs to triple bonds: mostly nitrosylation reactions, addition to nitrilium ions, and addition to diazonium ions.

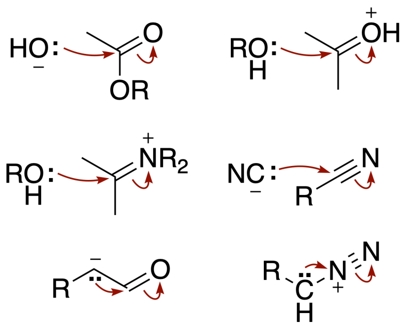

Figure S22: Examples of non-bonding lone pairs adding to π^* orbitals.

Most frequently, addition to conjugated systems was represented using all the atoms of the π^* system and less frequently using only the first two atoms (Fig. S23). The initial dataset contains only one example of lone pair addition to a 6-atom π^* acceptor, which was a pyridinium ion.

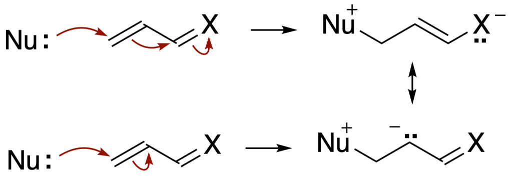

Figure S23: Two equivalent representations of conjugate addition.

C. Lone Pair Adding to σ^*

Over half of the initial dataset involved the addition of lone pairs to σ^* orbitals. Most of these mechanistic steps involve proton transfers from heteroatoms to heteroatoms, from acidic species such as carboxylic acids, alcohols, sulfonic acids, ammonium ions, iminium ions, and protonated heterocycles (Fig. S24). These types of proton transfers tend to be very facile.[3] Notably, proton transfers via strained 4-membered transition states were excluded with the expectation that intermolecular proton transfers via linear transition states would be more facile.[4] Close to a fourth of the mechanistic steps in this category involved the addition of heteroatom lone pairs on O, N, or O- into an adjacent σ^* orbital to push out a leaving group. This type of elimination process is a normal part of most acyl, sulfonyl, and phosphoryl substitution processes involving addition-elimination mechanisms. It is also the essential C–C bond cleaving step in retro-aldol-type processes. There are some examples of lone pair addition to halogenating agents with I–X, Br–X, and Cl–X bonds, but these types of electrophiles are more commonly partnered with soft π_{CC} nucleophiles. Simple bimolecular (S_N2) displacements not centered on hydrogen atoms were less common in the dataset.

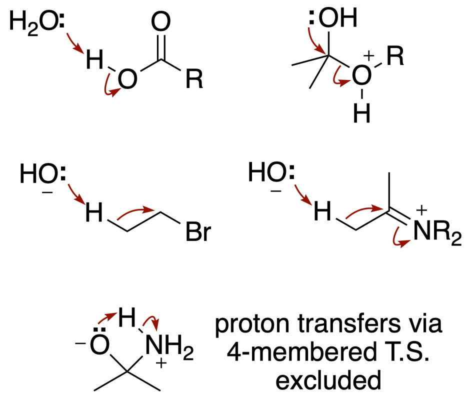

Figure S24: Addition of non-bonding lone pairs to σ^* electrophiles.

II. Source: π Bonds

A. π Bond Adding to Empty Orbital

Less than 3% of the initial dataset involved the addition of π nucleophiles to empty orbitals (Fig. S25). Carbon-carbon π bonds are generally viewed as soft nucleophiles that don't usually pair with hard electrophiles like carbenium cations. The most common arrow-pushing steps in this category were associated with electrophilic aromatic substitution processes where aromatic and heteroaromatic π systems add to the empty orbitals of carbenium ions and HO_3S^+ ions. Many other electrophilic aromatic substitution processes were represented in addition to π^* acceptors, such as acylium ions, and are discussed in the next section.

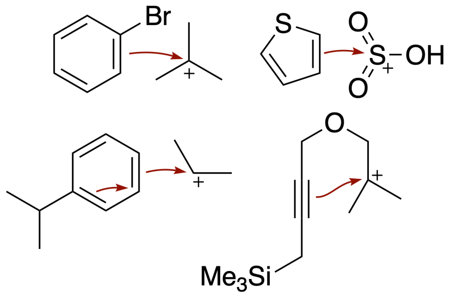

Figure S25: Examples of π nucleophiles adding to empty orbitals.

B. π Bond Adding to π^*

About 5% of the arrow-pushing steps in the initial dataset depict the addition of π bonds to π^* orbitals (Fig. S26) and about a fourth of these steps were simply resonance representations (e.g., $C{=}O \leftrightarrow {}^+C{-}O^-$). Most of the pi nucleophiles were double bonds as opposed to C≡C triple bonds or cumulenes – e.g., enols, enolates, enamines, allylsilanes, allylstannanes, and aromatic pi systems. There were few examples heteroatomic π bonds attacking π^* (e.g., diazoalkanes $R_2C{=}N^+{=}N^-$). About a fifth of the steps involving π bonds adding to π^* involved oxonium $C{=}O^+$ electrophiles, representing some of the most important C–C bond-forming processes in modern organic synthesis such as aldol reactions of lithium and boron enolates via 6-membered transition states, and Prins-type additions to $C{=}O^+$ ions. Many of the common electrophilic aromatic substitution processes

involve the addition of aromatic pi bonds to π^* electrophiles; for example nitration and Friedel-Crafts acylation. Over 5% of the steps in the π adding to π^* category involved the addition of enols, enolates, and enamines to iminium ions (C=N$^+$); iminium ion acceptors are important in Mannich and Knoevenagel reactions and are central to the field of organocatalysis. There were only a handful of examples of soft pi bonds adding to S=O or P=O, which are much more common acceptors for hard lone pair nucleophiles on oxygen or nitrogen.

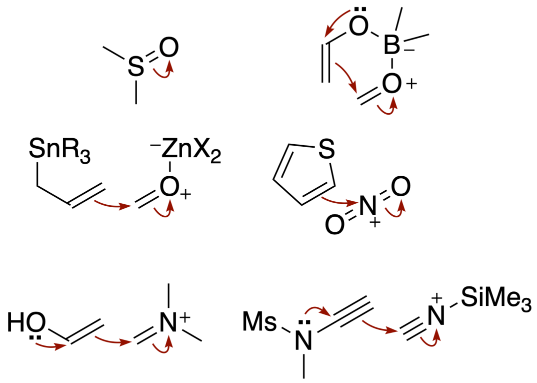

Figure S26: Examples of π nucleophiles adding to π^* electrophiles.

C. π Bond Adding to σ^*

The initial dataset involved a modest number (<7%) of arrow-pushing steps involving π bonds adding to σ^* orbitals (Fig. S27). About a fourth of these steps involved the protonation of C=C double bonds in enols, enolates, enamines, allysilanes, propargylsilanes and regular alkenes. Sulfuric acid was slightly over-represented as the acid in these cases because enols, enolates and enamines will rarely be present in the same reaction milieu with sulfuric acid. There were a few examples of heteroatomic pi bonds such as protonation of N-nitroso groups (R_2N–N=O). Protonation of P=O and S=O was depicted to use the oxygen lone pair and not the π bond. Over 10% of the π to σ^* steps involved halogenation of π_{CC}, including many examples of allylsilanes, vinylsilanes, and related groups. Beta-elimination of hydroxide, alkoxide, and halide groups in E1$_{CB}$ mechanisms were also part of this category. There were some examples of displacements with neighboring group participation to give cyclopropylcarbinyl and norbornyl cations.

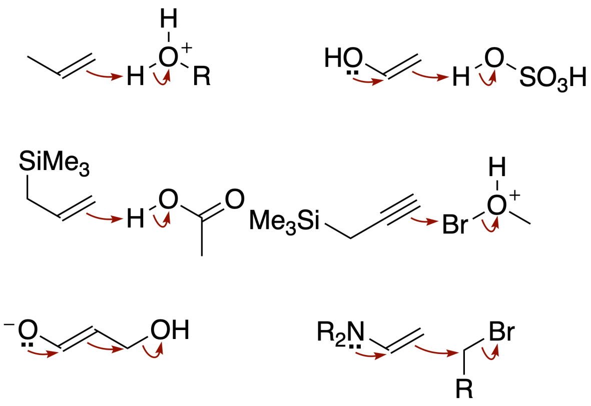

Figure S27: Examples of π orbitals adding to σ^*.

III. Source: σ Bonds

A. σ Bond Adding to Empty Orbital

Typical σ bonds are not very nucleophilic so there were only about 200 entries involving σ bonds adding to empty orbitals in the initial dataset (Fig. S28). The majority of these reaction steps were 1,2-shifts like Wagner-Meerwein and pinacol rearrangements. 1,2-Alkyl shifts are pericyclic but are included in this database of polar reaction steps because they can be described as a single nucleophile adding to a single electrophile. There were also examples of ionization of beta-silyl carbocations (and related metallocarbenium ions) to form π bonds. Both steps of metal-halogen and tin-lithium exchange were represented in this category: for example, the addition of a C–Li bond to form anionic R–I$^-$–R followed by the addition of a C–I$^-$ bond a Li$^+$ cation. There were relatively few examples of intermolecular addition of carbon–metal bonds to boron, aluminum, phosphonium groups.

B. σ Bond Adding to π^*

The initial dataset contained several hundred examples of σ bonds adding to double or triple bonds (Fig. S29). This group of arrow-pushing steps contained dozens of closely related examples of Meerwein-Ponndorf-Verley/Oppenauer hydride transfers. The remaining set of nucleophilic σ bonds were mostly hydridoborates , hydridoaluminates, or organometallics like organolithium and

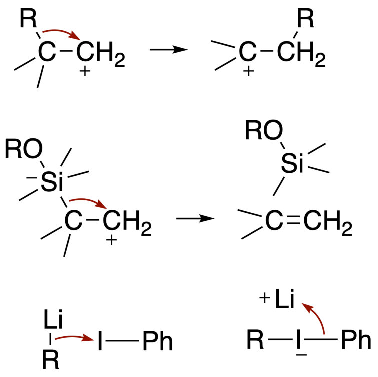

Figure S28: Examples of σ orbitals adding to empty orbitals.

Grignard reagents. About 90% of the π^* acceptors for σ bonds were carbonyl groups; oxonium ions (C=O$^+$) were more common than neutral carbonyls (C=O). Most of the other pi acceptors for σ bond nucleophiles were iminium ions and imines.

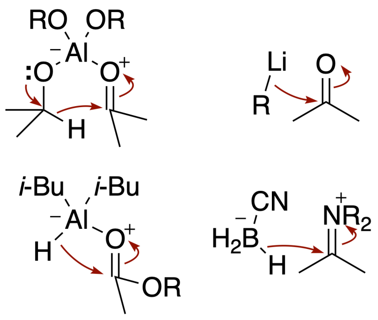

Figure S29: Examples of σ bonds adding to π^*.

C. σ Bond Adding to σ^*

The initial dataset contained almost a thousand examples of σ bonds adding to σ^* orbitals (Fig. S30A). About two-thirds of the mechanistic steps in this group are dissociative processes, like the first step in an S_N1 substitution mechanism (Fig. S30B) or expulsion from a pentavalent siliconate. It is not common to describe dissociative processes as a bond attacking itself, but it is consistent with the excellent spatial overlap between the σ and σ^* frontier orbitals. About a tenth of the steps in this category correspond to proton transfers. Examples of deprotonation by Na–H bonds were over-represented because NaH is a common base in sophomore textbooks. There were slightly fewer examples of protonation of carbon-metal bonds such as alkyllithium

and Grignard reagents, which are generally used under aprotic reaction conditions. There were dozens of examples of migratory displacements driven by a neighboring oxygen lone pair: pinacol rearrangements, Grob fragmentations, Tiffeneau-Demjanov reactions, Bayer-Villiger reactions, peroxyboronate migrations, etc.

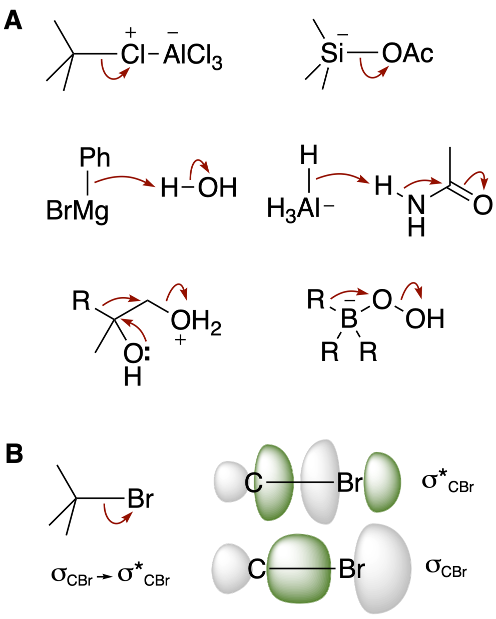

Figure S30: Examples of σ bonds adding to σ^*.

References

(1) Ruhlandt-Senge, K.; Henderson, K.; Andrews, P. C. *Compr. Organomet. Chem. III*; Elsevier, 2007; pp 1–65.

(2) Schmidt, P.; Lochmann, L.; Schneider, B. Structure and vibrational spectra of the sodium and potassium tert-butoxides. *J. Mol. Struct.* **1971**, *9*, 403–411.

(3) Crooks, J. *Compr. Chem. Kinet.*; Elsevier, 1977; Vol. 8; pp 197–250.

(4) Taylor, R.; Kennard, O. Hydrogen-bond geometry in organic crystals. *Acc. Chem. Res.* **1984**, *17*, 320–326.
